# The applicability of the 21-gene assay to inform chemotherapy benefit in lymph node positive hormone receptor positive male breast cancer

**DOI:** 10.1007/s10549-026-07978-6

**Published:** 2026-05-20

**Authors:** Anu G. Gaba, Li Cao, Rebecca J. Renfrew

**Affiliations:** 1 Sanford Roger Maris Cancer Center, Department of Medicine, University of North Dakota, 820 4th Street N, Fargo, ND 58102 USA; 2 Sanford Center for Biobehavioral Research, 120 8th St S, Fargo, ND 58103 USA; 3 Sanford Health, 820 4th Street N, Fargo, ND 58102 USA

**Keywords:** Oncotype DX assay, Recurrence score, Male breast cancer, National cancer database, Lymph node positive

## Abstract

**Purpose:**

Our study aimed to determine if chemotherapy administration based on the 21-gene Oncotype DX Recurrence Score (RS) in men with hormone receptor positive (HR+) Her2 negative (Her2-) lymph node positive(LN+) breast cancers (BC) impacted overall survival (OS).

**Patients and methods:**

We conducted a retrospective cohort study on adult men and women with HR+ Her2-, 1–3 axillary LN + BC, with a valid oncotype DX RS assay, diagnosed between the years 2004–2020, using the National Cancer Database. RS risk categories were defined as low risk: 0–13, intermediate risk:14–25, and high risk: ≥26.

**Results:**

Of the 77,820 patients included in the study, 900 (1.2%) were male and 76,920 (98.8%) were female. Higher RS (both as a continuous and categorical variable) was significantly associated with worse OS in males (*p* = 0.003 continuous, *p* = 0.006 categorical), females ≤ 50 years old and females > 50 years old (*p* < 0.001 both continuous and categorical, in both groups). In the stratified adjusted models, there was no association between receipt of chemotherapy and OS in males for all RS risk groups. Conversely, chemotherapy improved OS for women ≤ 50 and for women > 50 who belonged to the intermediate and high RS risk groups (*p* = 0.001 and *p* < 0.001 respectively (women ≤ 50); *p* = 0.005 and *p* < 0·001 respectively (women > 50)).

**Conclusion:**

RS as determined by the Oncotype DX assay is associated with OS in men and women with HR+ Her2- LN+ BC. However, RS is not an indicator of the benefit of chemotherapy on OS in men, in contrast to women, with HR+ Her2- LN+ BC.

**Supplementary Information:**

The online version contains supplementary material available at 10.1007/s10549-026-07978-6.

## Background

The Oncotype DX assay is based on the expression of 16 tumor associated genes and 5 reference genes which is then translated into a Recurrence Score (RS) with values ranging from 0 to 100, where a high RS indicates a higher risk of recurrence (ROR) [1]. The utility of the 21-gene Oncotype DX RS assay in predicting benefit from chemotherapy was first validated prospectively in lymph node negative(LN-) women, stage I and II, hormone receptor positive (HR+) Her2 negative (Her2-) breast cancer (BC) [2, 3], and subsequently in HR+ Her2- lymph node positive (LN+) women [4, 5].

The validity of the Oncotype DX RS assay was first studied in HR+ Her2- LN+ post-menopausal women in a retrospective analysis of tumor samples from patients recruited to the prospective Southwest Oncology Group (SWOG) S8814 trial, which had shown that chemotherapy with tamoxifen improved the survival benefit when compared to tamoxifen alone [4]. The retrospective analysis showed that some patients with lower RS did not benefit from the addition of chemotherapy. This led to a confirmatory prospective randomized trial, RxPONDER study, in pre- and post-menopausal HR+ Her2- LN+ (1–3 axillary LN+) women with BC, RS ≤ 25, to assess the benefit of chemotherapy in addition to antihormonal therapy (AHT) [5]. The RxPONDER study showed that pre-menopausal women (or women < 50) who were HR+ Her2- LN+ benefitted from chemotherapy for all values of RS [5]. In contrast, post-menopausal women (or women > 50) who were HR+ HER2- LN+ did not benefit from the addition of chemotherapy for RS ≤ 25. Most women with LN+ disease were treated with adjuvant chemotherapy prior to the RxPONDER study, but this study helped to change that practice.

Male BCs differ from female BCs in their biology, molecular profile and response to different AHT [6]. There are no prospective studies in men with BC and the applicability of the Oncotype DX assay. One retrospective study looked at RS results from Genomic Health in males and females and linked it to the National Cancer Institute’s Surveillance Epidemiology and End Results (SEER) database of patients who had Oncotype DX assay and showed that higher RS in men and women were associated with worse survival; and that men had worse survival than women for RS ≥ 31 [7].

Wang et al. looked at the National Cancer Database (NCDB) and assessed sex disparities in RS and mortality predictions in stage I and II BC patients and found that RS is associated with higher mortality in males at a lower threshold than that for females, up to RS 21 [8]. The mortality risk for men plateaued once RS exceeded 21, whereas in women mortality increased for RS > 23. Chemotherapy did not improve mortality among male patients with intermediate RS by both traditional (RS 18–30) and TAILORx cutoffs (RS 11–25). Their patient population was predominantly lymph node negative (81 and 83% LN- in males and females respectively). Additionally, they did not assess chemotherapy effect in low and high-risk RS categories for males.

The goals of our analysis were to determine if the Oncotype DX assay was applicable to men with HR+ Her2- LN+ BC and if RS cutoffs as shown in studies in women could be applied to men to determine if any male BC patients can be spared chemotherapy without impacting survival.

## Methodology

**Fig. 1:**
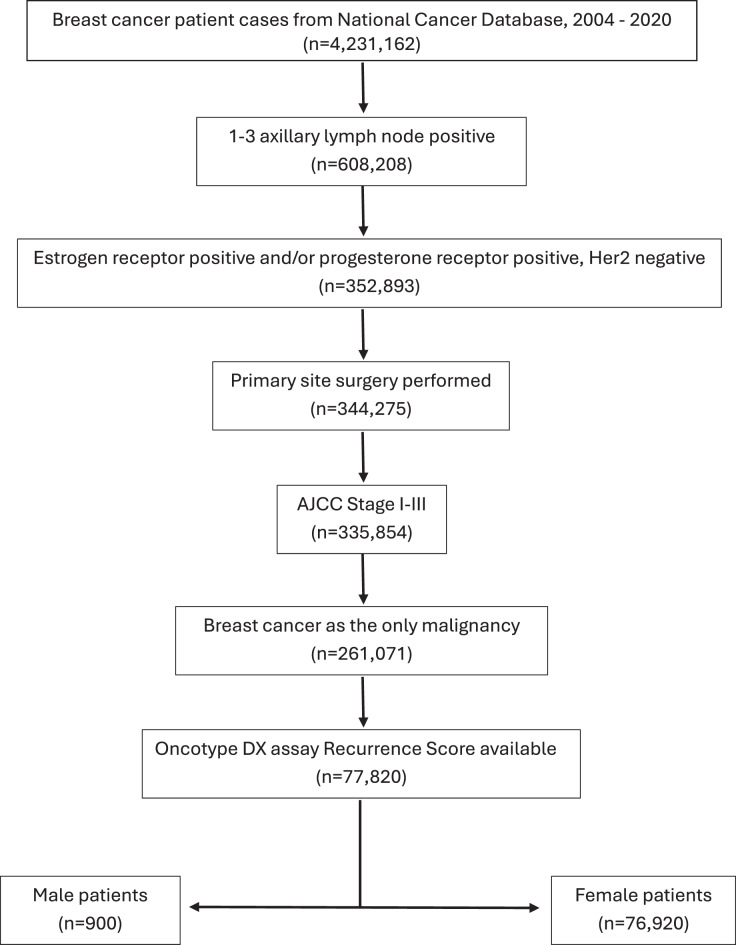
Patient selection flowchart. AJCC = American joint committee on cancer

We obtained data from the hospital-based National Cancer Database (NCDB) Participant User File (PUF), which is the largest database of records of patients with cancer in the US, capturing nearly 74% of newly diagnosed patients with cancer and approximately 82% of all new breast cancer cases diagnosed annually [9]. We requested records of all adult (≥18 years) BCs within the database diagnosed between the years 2004 and 2020. We selected male and female patients with Stage I, II, and III BCs which were HR+ Her2- and positive for 1–3 axillary LNs only (Fig. 1). Male and female categories were assigned based on the information available in the NCDB, as recorded from the medical record of each patient. Patients belonging to other non-binary sex categories were suppressed in the PUF and not available for this study. Additionally, the patients must have had a valid Oncotype DX Recurrence Score recorded. Stage at diagnosis was defined according to the then current American Joint Committee on Cancer (AJCC) staging manual at the time the cancer was diagnosed.

Given the retrospective nature of this study using de-identified patient level data, this study was exempt from patient consent and IRB approval, as per our institutional policy.

The main outcome measure was overall survival(OS). The sociodemographic data collected from the NCDB were age at diagnosis, race, insurance status, Charlson Deyo comorbidity score (CDS), and median income of the zip code of residence. The median income of the zip code of residence was defined as being estimated by matching the zip code of the patient’s residence recorded at the time of diagnosis against files derived from the American Community Survey data (United States Census Bureau). Categories 1–4 are quartiles based on equally proportioned income ranges among all US zip codes; 1 being the lowest quartile and 4 being the highest income quartile. The tumor characteristics that were collected were tumor size and grade, RS, number of regional lymph nodes positive, lympho-vascular invasion, and receipt of chemotherapy, hormone therapy, and radiation

The fields used within the NCDB dataset to determine OS were “Last Contact or Death, Months from DX” and “Vital Status.” Vital status was recorded as ‘dead’ or ‘alive’ and pertained to the status at the time of last contact.

Descriptive statistics were used to summarize demographic, clinical, and treatment variables by sex. Chi-square (χ^2^) tests compared categorical variables, and independent *t*-tests compared continuous variables. A two-sided *p*-value < 0.05 was considered statistically significant.

Cox proportional hazards model assessed the association between Recurrence Score (RS) and OS, analyzed as both continuous and categorical variables. RS risk categories that we used were low risk (0–13), intermediate risk (14–25), and high risk (≥26) as used in the RxPONDER trial [5]. Cox proportional unadjusted and adjusted models, stratified by sex and RS category, were used to assess the impact of chemotherapy on OS. The covariates used were race, lympho-vascular invasion, comorbidities, tumor grade, tumor size, year of diagnosis, age, income and insurance. All analyses were performed using SPSS Version 29.0 (IBM Corp., Armonk, NY). The R packages “survival” and “survminer” were applied to establish the OS figures.

## Results

### Descriptive characteristics

Of the 77,820 patients included in the study, 900 (1.2%) were male and 76,920 (98.8%) were female. The mean follow-up duration was slightly longer among female patients than male patients. − 65.9 months for females and 55.3 months for males (*p* < 0.001), while the median follow-up was 58.9 months (range 0–186 months) and 47.2 months (range 3–161 months), respectively. Table 1 summarizes the baseline characteristics. Males were older (89.1% vs. 75.7% aged > 50, *p* < 0.001), more likely to have government insurance (50.1% vs. 40.7%, *p* < 0.001), and had more comorbidities based on the CDS (7.9% vs. 4.2%, *p* < 0.001).

**Table 1:** Descriptive information of study subjects by sex

		Male	Female	p
		n = 900	n = 76,920	
Age	≤50	98(10.9)	18726 (24.3)	<0.001
	>50	802 (89.1)	58194 (75.7)	
Race	Non-White	167 (18.6)	12685 (16.5)	0.097
	White	733 (81.4)	64235 (83.5)	
Chemotherapy	No	631 (70.1)	53306 (69.3)	0.415
	Yes	258 (28.7)	23156 (30.1)	
	Missing	11 (1.2)	458 (0.6)	
Lympho-vascular Invasion	Absent or not identified	422 (46.9)	42966 (55.9)	<0.001
	Present or identifies	396 (44.0)	25215 (32.8)	
	Unknown or missing	82 (9.1)	8739 (11.4)	
Insurance	Private	422 (46.9)	43816 (57.0)	<0.001
	Government	451 (50.1)	31306 (40.7)	
	No insurance or missing	27 (3.0)	1798 (2.3)	
^1^Comorbidity	Yes CDS ≥ 1	71 (7.9)	3218 (4.2)	<0.001
	No CDS = 0	682 (75.8)	64226 (83.5)	
	Missing	147 (16.3)	9476 (12.3)	
Grade	I	80 (9.3)	16256 (21.2)	<0.001
	II	516 (60.0)	44067 (57.5)	
	III	264 (30.7)	12643 (16.5)	
	IV	0 (0.0)	17 (0.0)	
	Missing	35 (3.9)	3610 (4.7)	
RS Category	0–13	386 (42.9)	29750 (38.7)	<0.001
	14–25	328 (36.4)	35833 (46.6)	
	26+	186 (20.7)	11337 (14.7)	
RS	Mean (SD)	17.21 (11.36)	16.94 (9.65)	0.326
Regional Lymph Nodes	1	655 (72.8)	58213 (75.7)	0.014
	2	173 (19.2)	14242 (18.5)	
	3	72 (8.0)	4465 (5.8)	
Endocrine Therapy	Yes	785 (87.5)	71883 (93.6)	<0.001
	No	78 (8.7)	3981 (5.2)	
	Unknown or missing	34 (3.8)	921 (1.2)	
Radiation Therapy	Yes	556 (61.8)	58543 (76.1)	<0.001
	No	309 (34.3)	15869 (20.6)	
	Unknown or missing	35 (3.9)	2508 (3.3)	
Tumor Size	0–2 cm	369 (41.0)	40618 (52.8)	<0.001
	2–5 cm	446 (49.6)	28789 (37.4)	
	>5 cm	15 (1.7)	3470 (4.5)	
	Unknown or missing	70 (7.8)	4043 (5.3)	
^2^Income	1	86 (9.6)	8245 (10.7)	0.656
	2	157 (17.4)	12490 (16.2)	
	3	173 (19.2)	15500 (20.2)	
	4	345 (38.3)	28980 (37.3)	
	Missing	139 (15.4)	11705 (15.2)	

^1^The Charlson-Deyo Score (CDS) is a calculated data item that gives a weighted score to each patient based on comorbid conditions submitted to the NCDB as ICD-9-CM or ICD-10-CM codes. Select comorbid conditions are assigned a Charlson-Deyo Score. Each patient has a calculated Total Charlson-Deyo Score based on the number of and which comorbid condition the patient has. A score of 0 indicates that the patient did not have any of the selected comorbid conditions that are associated with a Charlson-Deyo Score. A score of 3 or more indicates that a patient has more of the select comorbid conditions or has the higher weighted comorbid conditions

^2^The median income of the zip code of residence was defined as being estimated by matching the zip code of the patient’s residence recorded at the time of diagnosis against files derived from the American Community Survey data (United States Census Bureau). Categories 1–4 are quartiles based on equally proportioned income ranges among ^3^all US zip codes; 1 being the lowest quartile and 4 being the highest income quartile

RS = Recurrence Score

Males had higher rates of grade III tumors (30.7% vs. 16.5%, *p* < 0.001) and lympho-vascular invasion (44.0% vs. 32.8%, *p* < 0.001). The mean RS were not significantly different between males and females (*p* = 0.326). In comparison to females, males were more likely to have a high RS (≥26) (20.7% vs. 14.7%) and low RS (0–13) (42.9% vs 38.7%), while intermediate RS (14–25) (36.4% vs 46.6%) was less frequent (*p* < 0.001).

Males were less likely to receive endocrine therapy (87.5% vs. 93.6%) and radiation (61.8% vs. 76.1%; *p* < 0.001 for both therapies).

## Survival analysis

Cox models showed RS was significantly associated with OS in both sexes when assessed as both continuous and categorical variables (Table 2). The results showed that higher RS was associated with worse OS, this was true for males (*p* = 0.003 continuous, *p* = 0.006 categorical), females ≤ 50 (*p* < 0.001 continuous, *p* < 0.001 categorical), and females > 50 (*p* < 0.001 continuous, *p* < 0.001 categorical).

**Table 2:** The association of the OncoType DX Recurrence Score assay (continuous scale and categorical) with overall survival by sex

			HR	95% CI		*p*-value
Male						
	RS continuous scale		1.025	1.009	1.042	0.003
	RS category					0.006
		<13	Reference			
		14–25	1.466	0.891	2.416	0.132
		26+	2.25	1.366	3.706	0.001
Female						
	RS continuous scale		1.03	1.027	1.032	<0.001
≤50 years old	RS category					<0.001
		<13	Reference			
		14–25	1.813	1.452	2.265	<0.001
		26+	3.919	3.297	4.657	<0.001
>50 years old	RS category					<0.001
		<13	Reference			
		14–25	1.202	1.12	1.291	<0.001
		26+	2.245	2.09	2.412	<0.001

RS = Recurrence Score

HR = Hazard Ratio

95% CI = 95% Confidence Interval

**Fig. 2:**
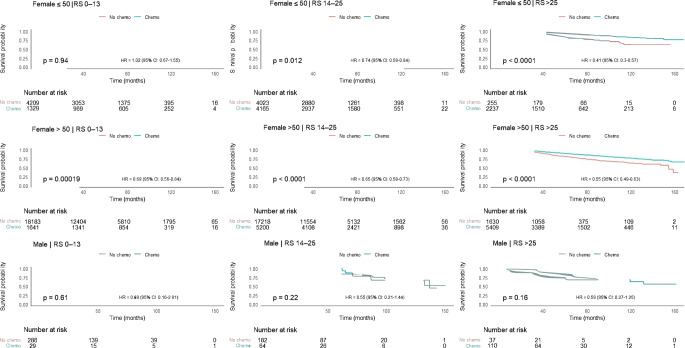
Association of chemotherapy with overall survival for males, females ≤ 50, females > 50, with hormone receptor positive, lymph node [1–3] positive, breast cancer for each Recurrence Score category (unadjusted). RS = Recurrence Score

In the stratified unadjusted models, there was no association between receipt of chemotherapy and OS in males for all RS groups (Table 3, Fig. 2). Chemotherapy improved the survival for women ≤ 50 years old who belonged to the intermediate and high RS groups (*p* = 0.012 and *p* < 0.001 respectively). Chemotherapy improved OS for females > 50 in all RS groups (*p* < 0.001for each of the three groups). The significant impact of chemotherapy on OS for the various RS categories remained the same even after adjusting for potential confounders – race, lympho-vascular invasion, comorbidities, tumor grade, tumor size, year of diagnosis, age, income and insurance,- except for women > 50 with RS 0–13, for whom chemotherapy no longer showed an association with OS. (The results of the univariate analysis of association of the above confounders with overall survival is depicted in Supplementary Table S1 and Figure S1)

**Table 3:** Association of overall survival with chemotherapy (unadjusted and adjusted) stratified by Recurrence Score for males, females ≤ 50, and females > 50

Unadjusted			HR	95% CI	*p*-value
Male					
	0–13	No Chemo	Reference		
		Chemo	0.69	0.16–2.91	0.61
	14–25	No Chemo	Reference		
		Chemo	0.55	0.21–1.44	0.22
	26+	No Chemo	Reference		
		Chemo	0.58	0.27–1.25	0.16
Female ≤ 50					
	0–13	No Chemo	Reference		
		Chemo	1.02	0.67–1.55	0.94
	14–25	No Chemo	Reference		
		Chemo	0.74	0.58–0.94	0.012
	26+	No Chemo	Reference		
		Chemo	0.41	0.30–0.57	<0.001
Female > 50					
	0–13	No Chemo	Reference		
		Chemo	0.69	0.56–0.84	<0.001
	14–25	No Chemo	Reference		
		Chemo	0.65	0.58–0.73	<0.001
	26+	No Chemo	Reference		
		Chemo	0.55	0.484–0.63	<0.001
Adjusted^1^			HR	95% CI	p-value
Male					
	0–13	No Chemo	Reference		
		Chemo	2.15	0.45–10.29	0.34
	14–25	No Chemo	Reference		
		Chemo	0.45	0.09–2.21	0.32
	26+	No Chemo	Reference		
		Chemo	0.92	0.32–2.67	0.88
Female ≤ 50					
	0–13	No Chemo	Reference		
		Chemo	0.81	0.48–1.37	0.43
	14–25	No Chemo	Reference		
		Chemo	0.62	0.47–0.83	0.001
	26+	No Chemo	Reference		
		Chemo	0.34	0.23–0.51	<0.001
Female > 50					
	0–13	No Chemo	Reference		
		Chemo	0.85	0.66–1.10	0.22
	14–25	No Chemo	Reference		
		Chemo	0.82	0.71–0.94	0.005
	26+	No Chemo	Reference		
		Chemo	0.51	0.52–0.73	<0.001

^1^Analysis was adjusted for the following covariates: race, lympho-vascular invasion, comorbidities, tumor grade, tumor size, year of diagnosis, age, income, and insurance

## Discussion

In our large retrospective study using the NCDB, we found that males with LN + HR+ breast cancer who had the oncotype DX assay had mean RS scores similar to LN+HR+ females. But males were more likely to have RS in the low risk and high risk categories, unlike females who were more likely to have RS in the intermediate risk category. This distribution pattern was similar to that seen in the Wang study which looked at predominantly LN-HR+ breast cancer [8]. Altman et al. also showed that higher proportion of men had high risk RS as compared to women, and these men were more likely to have grade 3 tumors and PR negative disease [10]. Similar distribution of higher proportion of men in the high and low risk RS categories as compared to women was seen in a study done using oncotype DX assay results from genomic health between the years 2004–2017 [7]. This study also showed that proliferation gene groups and invasion gene groups were higher in men than in women, so also the ER gene expression. Male breast cancers have a higher proportion of germline BRCA1 and 2 mutations compared to females [11–14]. Studies have also shown difference in molecular make up of male breast cancers, and these could reflect on the differences in the distribution of the RS between men and women [7, 15, 16].

It is well known that RS is an independent predictor of disease recurrence, both locoregional and distant, in both LN+ and LN- HR+ breast cancer in women [17, 18]. Wang’s retrospective study on LN- HR+ breast cancers also showed that higher RS correlated with OS in both men and women [8]. Our findings too confirmed that women with higher risk RS had worse OS. These findings held true with RS as both a continuous and categorical variable. However, in men, although higher RS on a continuous scale was significantly associated with worse OS; when RS was analyzed as a categorical variable, it was only men high risk RS that had worse OS when compared to men with low risk RS. There was no difference in OS between the low and intermediate risk RS groups. Our study differed from the Wang study, in that all our patients had LN+ disease.

Our analysis showed that there was a significant association between receipt of chemotherapy and survival in the intermediate and high RS risk categories in women ≤ 50 and in women > 50. The outcomes in premenopausal and post-menopausal women are only partially in line with the Kalinsky study which showed that both low and intermediate risk RS categories, LN+ premenopausal women do benefit from chemotherapy in contrast to LN+ postmenopausal women, where there is no benefit of chemotherapy [5]. The reason for these seemingly different results between the Kalinsky study and our study, is the different endpoints; the endpoints of the Kalinsky study- invasive disease free survival and distant relapse-free survival whereas our study used overall survival. Nevertheless, unlike in women, there was no impact of chemotherapy on OS in LN+ men for any of the RS categories in our study. This was true in both unadjusted models as well as when the analysis was repeated after adjusting for – race, lympho-vascular invasion, comorbidities, tumor grade, tumor size, year of diagnosis, age, income and insurance. In Wang’s study, where the population was predominantly men who were HR+ Her2- and LN negative, chemotherapy did not improve mortality among male patients with intermediate RS by both traditional (RS 18–30) and TAILORx (RS 11–25) risk categories [8]. They did not assess chemotherapy impact in the low and high risk RS categories.

One retrospective SEER based analysis found an OS benefit for chemotherapy vs. no chemotherapy in HR+ Her2- men with Stage I,II and III breast cancer [19]. However, on subset analysis, there was no benefit for chemotherapy in LN negative men irrespective of tumor size, and no benefit in LN+ men for tumors less than 2 cm. This contradicts with our study, which showed no survival benefit for chemotherapy in any of the RS risk categories in our patient population, which was men with HR+Her2- LN+ breast cancer and more than 99% had tumor size greater than 2 cm. One reason for this difference is that our patients were selected for those men who had undergone an oncotype DX assay; clinical characteristics of this patient population may not completely match the subset in Li’s study that did demonstrate an OS benefit for the use of chemotherapy. Our study excluded patients with greater than 3 positive LNs, since those would not have fit in with the Kalinsky criteria per RXPONDER study [9].

Our study is unique in that it gives a first glimpse into the absence of the impact of chemotherapy, based on RS, impacting outcomes in 1–3 LN+ HR+HER2- men with breast cancer. Even though higher RS predicted worse overall survival in men, chemotherapy was not associated with improved overall survival in any of the RS categories in our population based retrospective study. Currently, decisions to treat men with chemotherapy are based on risk factors including RS, obtained from data extrapolated from women. Our findings caution us against using this approach. Prospective studies in men questioning the predictive value of oncotype DX assay as a deciding factor for chemotherapy administration are needed.

The strengths of our study are the large sample size of male patients with HR+Her2- LN+ breast cancer. One limitation of this study was the unavailability of breast cancer recurrence data in the NCDB. Hence we could not measure time to local or distant recurrence or recurrence free survival. Our main end point had to be overall survival. We had a disproportionate number of patients in the two arms of the study due to males having a lower incidence of BC, yet the NCDB remains one of the best resources available to gather detailed information on as many male BCs as possible.

## Conclusion

In our large retrospective analysis, RS as determined by the Oncotype DX assay is associated with OS in men and women with HR+ Her2- LN+ BC. However, RS is not an indicator of the benefit of chemotherapy on OS in men, in contrast to women, with HR+ Her2- LN+ BC. Prospective studies are needed in male breast cancer patients, to establish the benefit of incorporation of the Oncotype DX assay to determine if chemotherapy can impact outcomes when added to endocrine therapy.

## Electronic supplementary material

Below is the link to the electronic supplementary material.

Supplementary material 1

Supplementary material 2

## Data Availability

Data was used from the The National Cancer Database (NCDB) Participant User Data File (PUF) after submitting an application. The NCDB PUF is a Health Insurance Portability and Accountability Act (HIPAA)-compliant data file containing cases submitted to the Commission on Cancer’s (CoC) NCDB. The PUF contains de-identified patient level data.
