# Supplementary material for: The applicability of the 21-gene assay to inform chemotherapy benefit in lymph node positive hormone receptor positive male breast cancer
Source: Breast Cancer Res Treat. 2026 May 20;217(2):36. doi: 10.1007/s10549-026-07978-6 (PMC13190426; doi:10.1007/s10549-026-07978-6)
Supplement: Supplementary file 1 — Supplementary material 1 [file 10549_2026_7978_MOESM1_ESM.docx]

Supplementary Table S1. Univariate Analysis of Association of Covariates with Overall Survival

| Variables |  | HR | 95 % CI | p-value |
| --- | --- | --- | --- | --- |
| Insurance |  |  |  |  |
|  | No insurance | Reference |  | <0.001 |
|  | Private | 0.646 | 0.532-0.785 |  |
|  | Government | 1.783 | 1.472-2.161 |  |
| Race |  |  |  |  |
|  | Non- White | Reference |  | 0.334 |
|  | White | 0.962 | 0.889-1.041 |  |
| ^1^CDS |  |  |  |  |
|  | No CDS=0 | Reference |  | <0.001 |
|  | Yes CDS>=1 | 2.307 | 2.168-2.456 |  |
| Grade |  |  |  |  |
|  | 1 | Reference |  | <0.001 |
|  | 2 | 1.347 | 1.245-1.457 |  |
|  | 3 | 2.443 | 2.236-2.669 |  |
|  | 4 | 1.729 | 0.557-5.374 |  |
| Lympho-Vascular Invasion | |  |  |  |
|  | Absent or not identified | Reference |  | <0.001 |
|  | Present or identified | 1.344 | 1.263-1.429 |  |
| Year of Diagnosis | |  |  | <0.001 |
|  |  | 1.023 | 1.011-1.035 |  |
| Tumor size |  |  |  |  |
|  | ≤ 2 cm | Reference |  | <0.001 |
|  | 2-5 cm | 1.902 | 1.791-2.02 |  |
|  | > 5 cm | 2.182 | 1.921-2.477 |  |
| Age at Diagnosis | |  |  | <0.001 |
|  |  | 1.071 | 1.046-1.096 |  |
| ^2^Income |  |  |  |  |
|  | 1 |  |  | <0.001 |
|  | 2 | 0.86 | 0.780-0.948 |  |
|  | 3 | 0.71 | 0.646-0.782 |  |
|  | 4 | 0.528 | 0.483-0.578 |  |

^1^The Charlson-Deyo Score (CDS) is a calculated data item that gives a weighted score to each patient based on comorbid conditions submitted to the NCDB as ICD-9-CM or ICD-10-CM codes. Select comorbid conditions are assigned a Charlson-Deyo Score. Each patient has a calculated Total Charlson-Deyo Score based on the number of and which comorbid condition the patient has. A score of 0 indicates that the patient did not have any of the selected comorbid conditions that are associated with a Charlson-Deyo Score. A score of 3 or more indicates that a patient has more of the select comorbid conditions or has the higher weighted comorbid conditions.

^2^ The median income of the zip code of residence was defined as being estimated by matching the zip code of the patient’s residence recorded at the time of diagnosis against files derived from the American Community Survey data (United States Census Bureau). Categories 1-4 are quartiles based on equally proportioned income ranges among all US zip codes; 1 being the lowest quartile and 4 being the highest income quartile.
