# Supplementary material for: The applicability of the 21-gene assay to inform chemotherapy benefit in lymph node positive hormone receptor positive male breast cancer
Source: Breast Cancer Res Treat. 2026 May 20;217(2):36. doi: 10.1007/s10549-026-07978-6 (PMC13190426; doi:10.1007/s10549-026-07978-6)

**Supplementary Figure S1.** Forest Plot Showing Univariate Analysis of Association of Covariates with Overall Survival in hormone receptor positive, lymph node (1-3) positive, breast cancer.


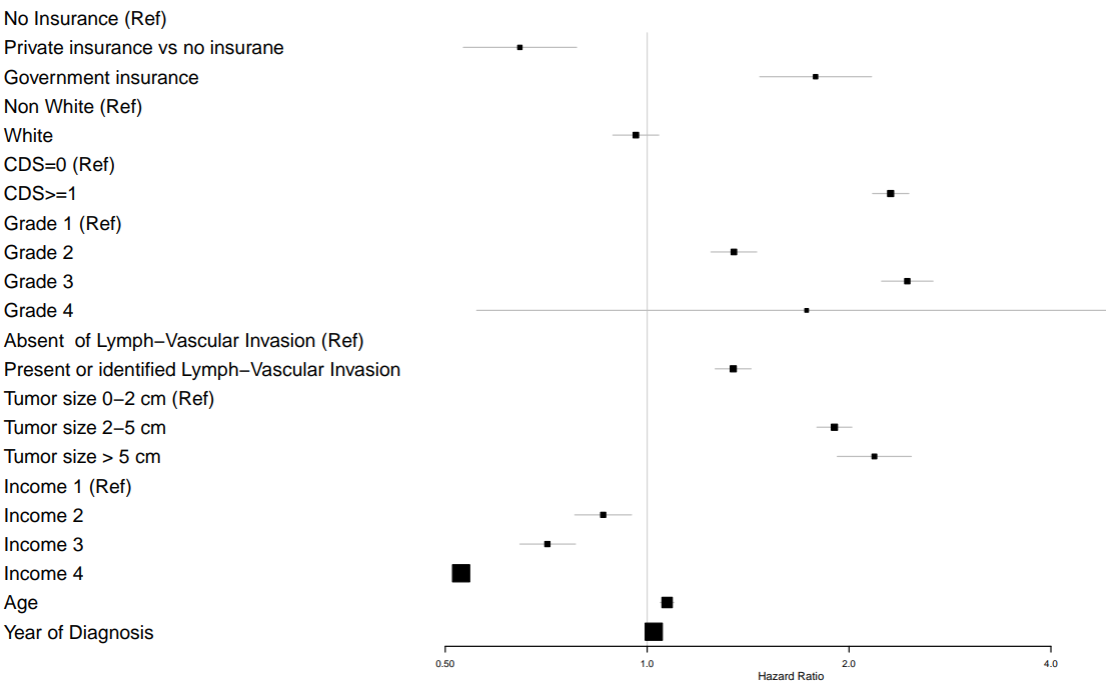

Supplement: Supplementary file 2 — Supplementary material 2 [file 10549_2026_7978_MOESM2_ESM.docx]
